# Clinical Parameters and Epigenetic Biomarkers of Plaque Vulnerability in Patients with Carotid Stenosis

**DOI:** 10.3390/ijms23095149

**Published:** 2022-05-05

**Authors:** Laia Carballo-Perich, Dolors Puigoriol-Illamola, Saima Bashir, Mikel Terceño, Yolanda Silva, Carme Gubern-Mérida, Joaquín Serena

**Affiliations:** 1 Cerebrovascular Pathology Research Group, Girona Biomedical Research Institute (IDIBGI), RICORS-ICTUS, Parc Hospitalari Martí I Julià, Edifici M2, 17190 Salt, Spain; lcarballo@idibgi.org (L.C.-P.); dpuigoriol@idibgi.org (D.P.-I.); 2 Cerebrovascular Pathology Research Group, Stroke Unit, Department of Neurology, Girona Biomedical Research Institute (IDIBGI), Dr. Josep Trueta University Hospital, RICORS-ICTUS, Av. França s/n (7a Planta), 17007 Girona, Spain; sbashir.girona.ics@gencat.cat (S.B.); mterceno.girona.ics@gencat.cat (M.T.); jserena.girona.ics@gencat.cat (J.S.)

**Keywords:** biomarker, carotid artery stenosis, stroke, neuroimaging, epigenetics, vulnerable plaque, atherosclerosis

## Abstract

Atheromatous disease is the first cause of death and dependency in developed countries and carotid artery atherosclerosis is one of the main causes of severe ischaemic strokes. Current management strategies are mainly based on the degree of stenosis and patient selection has limited accuracy. This information could be complemented by the identification of biomarkers of plaque vulnerability, which would permit patients at greater and lesser risk of stroke to be distinguished, thus enabling a better selection of patients for surgical or intensive medical treatment. Although several circulating protein-based biomarkers with significance for both the diagnosis of carotid artery disease and its prognosis have been identified, at present, none have been clinically implemented. This review focuses especially on the most relevant clinical parameters to take into account in routine clinical practice and summarises the most up-to-date data on epigenetic biomarkers of carotid atherosclerosis and plaque vulnerability.

## 1. Introduction

Cardiovascular diseases (CVDs) are the cause of approximately 58% of deaths and are responsible for more deaths than cancer, respiratory diseases, and diabetes taken together [1,2]. According to WHO data, stroke is the first cause of disability in adults, the second cause of dementia, the second cause of death in adult males, and the first cause of death of adult women in Western countries [3].

The social repercussions of CVDs are great: 32% of the deaths occur prematurely and are the most significant cause of long-term morbidity and disability. Stroke is responsible for 23% of years of life that are lost and 50% of years that are lived with disability. All of this not only has an enormous social cost but also an economic one. In our country, Spain, the cost represents 3–4% of the whole of health care expenditure, having a direct cost of 5000 €/patient/year and an average total of 20,000–40,000 €/patient/year, taking into consideration indirect costs [2]. If CVDs could be reduced, life expectancy would increase by an average of seven years, which would be lived with a better quality of life.

Atherosclerosis is a systemic inflammatory disease that mainly affects medium and large arteries such as the aorta, carotid, iliac, and coronary arteries, which are the main cause of CVDs and which are manifested as stroke, ischaemic cardiopathy, and peripheral artery disease. Atherosclerosis is associated with age and the presence of diverse classical vascular risk factors (VRFs) including: hypertension, dyslipidaemia, diabetes, tobacco use, obesity, and a sedentary lifestyle [4]. Despite the relevance and impact that its identification and control has on primary and secondary prevention of vascular events, the predictive capacity still needs improvement. The Framingham study found that between a third and half of CVDs are not due to classical VRFs [5]. Given this situation, studies aimed at identifying emerging VRFs have taken on great importance.

### Carotid Stenosis: Relevant but Not Sufficient to Identify Vulnerable Plaques

A total of 20–30% of ischaemic strokes of known cause are due to atherothrombotic disease, mainly due to carotid stenosis (10–20%), which is typically associated with the progressive growth and rupture of atheromatous plaques [6].

A transient ischaemic attack (TIA) precedes a disabling stroke in up to 25% of patients with carotid artery stenosis (CAS), offering an excellent opportunity for prevention if a rapid and adequate evaluation is performed. Through aetiological subgroups, the risk of recurrence is especially high in stroke with an atherothrombotic mechanism, with the observed benefit being highly dependent on the degree of stenosis for both a first ischaemic stroke and recurrence in these patients [7,8,9,10].

Carotid endarterectomy and carotid stenting are the standard revascularisation treatments for symptomatic carotid artery stenosis (SCAS) of >70% after suffering a TIA or minor stroke [11], with a lower benefit in SCAS of 50–70%, marginal utility in asymptomatic carotid artery stenosis (ACAS) >70%, and no clear benefit in ACAS of 50–69% or ACAS in women or older patients (>75 years).

The benefit in each of these scenarios is easy to compare if we consider the number of patients needed to treat to avoid an ipsilateral stroke in one year (NNT). In patients with SCAS >70%, SCAS 50–70%, and ACAS, the NNTs are 6–7, 15 and >150, respectively [7,8,9].This benefit in surgical treatment could be even lower nowadays if we consider recent advances in secondary stroke prevention with the introduction of angiotensin-converting enzyme (ACE) inhibitors, statins, and better compliance in the control of classical vascular risk factors [12,13]. Furthermore, most patients with CAS may not only not receive net benefit from endarterectomy or stenting, but are also submitted to the ever present risks associated with surgical procedures, making it highly desirable to improve the selection criteria.

To date, plaque vulnerability has rarely been taken into account in clinical decision making [14]. The term vulnerable plaque is used to refer to plaques that are susceptible to complications and their identification is a means of identifying apparently healthy subjects at risk of future ischaemic events. These vulnerable plaques can be thrombosis-prone or plaques with a high probability of undergoing rapid progression [15]. A variety of different processes are involved in plaque destabilisation including inflammation, lipid accumulation, apoptosis, proteolysis, thrombotic processes, and angiogenesis. While stable plaques show small necrotic cores, thick fibrous caps, and a calcified state, vulnerable plaques usually have a thin fibrous cap, a lipid-rich necrotic core, microscopic calcium deposits, and are characterised by increased inflammatory processes, macrophage infiltration, and neovascularisation [14,16]. In addition to the local features characterising plaque vulnerability, the evidence suggests that systemic factors may play a role in plaque instability including the presence of a systemic inflammatory state (vulnerable blood) [17]. In the field of coronary artery disease (CAD), the concept of the vulnerable patient, defined as a patient with high atherosclerotic burden, high-risk/vulnerable plaques, or vulnerable/thrombogenic blood, has been proposed [15,18,19].

In summary, although the degree of stenosis is an essential aspect in determining whether or not surgical treatment is indicated in both symptomatic and asymptomatic carotid stenoses, it is not in itself sufficient to evaluate the risk of future stroke in a significant number of patients. In the NASCET and ECST studies, it was observed that up to 40% of strokes during follow-up occurred in patients with CAS <50% whereas up to 80% of patients with CAS >50% did not suffer any stroke during the five years of follow-up [6,9,20]. An even more significant situation is seen in ACAS patients, where additional markers of plaque vulnerability need to be taken into consideration before surgical treatment can be indicated [21].

Biomarkers must present a specific measurable change that can be clearly associated with a diagnosis or a predictable outcome, providing information to physicians when evaluating the probability of developing a disease, making a diagnosis, and evaluating the severity of a disease and its progression, during therapeutic decision making or when monitoring a patient’s response [22]. Therefore, ideally, putative biomarkers should have diagnostic specificity and sensitivity, be able to differentiate between clinical subtypes, correlate with a specific outcome, provide prospective validation, have an incremental value with regard to existing markers, be clinically useful, and be rapidly quantified using cost-effective methodologies [23].

It would be clinically useful to detect serum/plasma markers associated with carotid plaque instability that would permit the identification of high and low risk patients, making it possible to better select patients requiring surgical or intensive medical treatment. The circulating concentration of vulnerable plaque biomarkers may increase (1) as a consequence of the rupture or progression of a vulnerable plaque or (2) due to pathological states—the aforementioned concept of vulnerable blood—that contribute to a high susceptibility to the development of vulnerable atherosclerotic plaques.

To date, several circulating protein-based biomarkers related to inflammation, lipid factors, metabolism, angiogenesis, and thrombosis with significance for both the diagnosis of CAS and its prognosis have been described and extensively reviewed [16,24,25]. However, at present, none of these have been clinically implemented to help physicians select patients at high risk of stroke who may benefit from surgical intervention. This suggests that new methodological strategies must be employed to search for markers of vulnerable plaques and, in this respect, epigenetic biomarkers have been gaining ground in the scientific community as tools for the diagnosis and prognosis of CVDs [22].

This review discusses the potential utility of clinical, haemodynamic, and neuroimaging data available in our routine clinical practice and provides an up-to-date summary of the state of knowledge on epigenetic biomarkers of carotid atherosclerosis and plaque vulnerability.

## 2. Clinical, Haemodynamic and Neuroimaging Parameters for Identifying Stroke Risk Subgroups

Table 1 summarises the main clinical, haemodynamic, and neuroimaging parameters described in the following subsections that may be useful in clinical practice.

### 2.1. Clinical Considerations in Risk Stratification

Surgical morbidity and mortality resulting from carotid endarterectomy is not negligible (5.8%) and the risk/benefit margin with respect to medical treatment is narrow, particularly in ACAS patients in whom carotid artery revascularisation should only be considered in patients with a life expectancy longer than five years and only if surgical morbidity and mortality of less than 3% can be achieved in order to obtain a net benefit of revascularisation over current medical treatment [7,8,9,10,11].

Along with the purely haemodynamic aspects that will be discussed below, some clinical elements have been identified that may be of help in cases where the optimal treatment is in doubt.

As a general rule, patients with SCAS >50% (NNT 15) and ACAS >70% (NNT >150) should be considered as candidates for endarterectomy or stenting. Within this general assessment, aspects such as the time elapsed since the clinical event (the risk of recurrence after three months is similar to that of asymptomatic patients), comorbidity, age, collateral circulation status, cerebrovascular reactivity (CVR), and the progression of the degree of stenosis should be considered.

Among the commonly used clinical parameters that have been shown to be associated with a high risk of stroke recurrence are male sex, diabetes (HR 1.82 (1.8–2.80)), and hemispheric stroke, particularly if the symptoms are suggestive of a haemodynamic origin (limb-shaking, precipitation of symptoms by exertion, exercise, or low blood pressure, retinal claudication) (HR 3.8, 95% CI 1.5 to 9.5), whereas a lower risk profile has been associated with moderate stenosis in women suffering a TIA, eye symptoms only [HR, 95% CI, 0 (0.0–0.6)], particularly in diabetic (NNT 60–80) and elderly patients [26,27,28].

Some factors are both high surgical risk factors (intraoperative or perioperative complications) and of surgical benefit (high risk of recurrence in medical treatment). As is usual in evaluating SCAS patients, the existence of a high surgical risk should not imply the exclusion of revascularisation treatment if the benefit outweighs the risk, particularly if endovascular treatment is considered to reduce the perioperative risk.

### 2.2. Haemodynamic Considerations in Risk Stratification

The variability in the anatomy of the polygon of Willis, which presents variants in up to 80–85% of the population (agenesis, hypoplasia, foetal circulation), determines the usual pattern of collateral circulation and its efficiency. Faced with the same degree of critical CAS (e.g., >80%), the stroke risk will be different depending on the CVR in each particular patient. CVR can be studied using transcranial Doppler (TCD) by determining the increase in blood flow in response to vasodilatory stimuli such as increased inspired carbon dioxide, intravenous injection of the carbonic anhydrase inhibitor acetazolamide and by the standardised apnoea test [29]. Impaired CVR reflects a collateral circulation failure and is associated with a risk of subsequent ipsilateral stroke, that is, between five and 10 times higher than in patients with preserved CVR [30,31,32,33]. This makes it one of the most useful tools in decision-making in patients in whom the indication for medical or surgical treatment is unclear.

In addition to the evaluation of the CVR, the TCD study, with or without an echo-contrast agent, allows a reliable assessment of the state of the intracranial vessels and collateral circulation including asymmetries between the middle cerebral arteries and efficient collateral flow through the anterior or posterior communicating arteries. The presence of collateral blood flow is associated with an increased stroke risk both in the anterior (HR 4.1, 95% CI 1.3 to 13.1) [32] and vertebrobasilar territories [34]. Probably one of the most significant elements in the suspicion of a CAS is the exploration of the ophthalmic artery, which is easy to evaluate through ultrasonography using an ophthalmic window. The detection of an inverted ophthalmic artery flow pattern allows us to affirm that there is a critical stenosis or occlusion of the ipsilateral carotid artery in the surgical range (>70%) and almost certainly >80%, with a failure of collateral circulation. Such patients are at high risk of suffering a first stroke or recurrent stroke and the risk/benefit balance is usually in favour of revascularisation.

Finally, the presence of asymptomatic microembolic signals (MES), detected by TCD, predicts subsequent ipsilateral stroke and thus suggests that TCD might be useful in identifying ACAS patients who have an increased risk of stroke or TIA and SCAS patients with a low risk and for whom surgical intervention would not be beneficial (HR 6.56, 1.60–26.86) [35].

### 2.3. High Risk Features of Atheromatous Plaque in Neuroimaging

While a significant minority of reports mention specific plaque features, some are high risk carotid markers that can be reliably assessed and reported by magnetic resonance imaging (MRI), computed tomography angiography (CTA), or ultrasound (US) in clinical practice with little additional effort [36].

An increased risk of ischaemic stroke has been associated with greater plaque volume, the progression of plaque volume over time, and its morphology [20]. Plaque thickness, and particularly plaque thickness >3 mm on CTA, is found to be more frequently associated with SCAS [37]. Other markers of plaque morphology associated with increased stroke risk are irregular surface of the atheromatous plaque and the presence of ulcerations as well as hypo/anechoic characteristics and intraplaque haemorrhage [20,36,37,38,39,40,41]. In contrast, the presence of calcified plaques is associated with a lower risk of stroke [36,41].

## 3. Epigenetic Biomarkers

Progress in the field of epigenetics has opened up a new world in the comprehension and management of human diseases including CVDs, based on the role of genetics and its environmental interaction [22]. Extensive epigenetic modifications contribute to atherosclerotic plaque development and progression, relying on the modification of DNA accessibility and structure without affecting DNA sequence, which are either heritable or temporary features [42]. DNA and histone proteins comprise the chromatin, which can be remodelled into a tightly condensed state (heterochromatin) or an open conformation (euchromatin) that would allow access to transcription factors or DNA binding proteins, which control gene expression [22].

Continuous efforts have been made to delineate the mechanisms beyond the atherosclerosis disease. Various risk factors for CVDs such as diabetes mellitus, nutrition, smoking, stress, hypertension, and circadian rhythm, often manifest as modifications of epigenetic marks, suggesting a crucial role for epigenetics in the regulation of the intrinsic genetic landscape and extrinsic environmental influences [43,44]. The reversible nature of epigenetic modifications makes it possible to regulate and reverse some phenotypes [43] such as plaque vulnerability, suggesting a potential clinical application in disease stratification or treatment [42]. In accordance with this idea, recent studies have focused on the potentially vulnerable plaques that exhibit remarkable plasticity and can change their status in response to local environmental cues, although the regulation of these processes at the epigenetic level remains largely unexplored [44].

The three main epigenetic modifications can be broadly categorised into DNA methylation, histone post-translational modifications (PTMs), and non-coding RNAs (ncRNAs). The current state of knowledge regarding all these epigenetic modifications as potential biomarkers of atherosclerosis, carotid stenosis, and plaque vulnerability is described in the following subsections and summarised in Supplementary Table S1.

### 3.1. DNA Methylation

DNA methylation is the most well-known epigenetic DNA modification and is an important mechanism of transcriptional regulation. Methylation at 5-cytosine (5-mC) is essential for proper gene expression, transposon silencing, alternative splicing, and genome stability [43]. However, it can be modified to 5-hydroxy methyl cytosine (5-hmC) by ten-eleven translocase enzyme (TET), mediating DNA demethylation. The DNA methylome undergoes programmed changes during cellular differentiation, but can also be modified by exogenous stimuli such as the diet and environmental factors, some of which coincide with known atherosclerosis risk factors [45]. Indeed, a link between peripheral blood cell DNA methylation profiles of specific loci to hyperlipidaemia, hyperglycaemia, and obesity has been described, so uncovering candidate circulating epigenetic markers of atherosclerosis and metabolic diseases predisposing to atherosclerosis [45,46,47]. In addition, it has been hypothesised that proatherogenic stimuli such as low-density lipoprotein (LDL) cholesterol and oxidised LDL may stimulate long-term epigenetic reprogramming of innate immune system cells, playing an important role in plaque development and vulnerability, and highlighting the importance of epigenetic biomarkers as predictors of CVDs [22].

Differentially methylated sites mapping to genes participating in atherogenesis have been identified by candidate gene-based studies and in an epigenome-wide survey of normal and atherosclerotic human aortic tissue, which showed a genome-wide increase in DNA methylation in atherosclerosis [45,48,49]. However, several controversies exist in DNA methylation, as both hypo- and hypermethylation have been associated with atherosclerotic lesions in humans and animal models [45,48]. For instance, Li et al. revealed that DNA hypomethylation sites were twice as numerous as hypermethylated sites in symptomatic plaques, suggesting that hypomethylation is predominant in carotid vulnerable plaques. Specifically, 14,657 genes were differentially methylated, of which 67% displayed hypomethylation in symptomatic plaques and were found to be involved in various aspects of inflammation [44]. Moreover, weak changes in the DNA methylome distinguish symptomatic from asymptomatic carotid plaques, but a widespread demethylation resulting in permissive transcriptional marks at atheroprotective gene promoters is established in plaques after a cerebrovascular event, supporting the idea that ruptured plaques tend to revert to a stable structure [45]. In contrast, promoter regions of atheroprotective genes such as oestrogen receptor 1/2 (ESR1/2), ATP-binding cassette transporter A1 (ABCA1), and kruppel-like factor 4 (KLF4) are often hypermethylated in atherosclerosis [43,48,50] and Kim et al. demonstrated higher methylation levels of genes involved in plaque progression and vulnerability such as autoimmune regulator 1 (AIRE1) and arachidonate 15-lipoxygenase (ALOX12), among others, in plaques than in non-plaque intima [51].

In line with this, it is strongly suggested that inflammation and immune activity are subjected to epigenetic regulation and involved in driving lesion progression and plaque destabilisation [44,52]. In fact, recent studies have described hypomethylation in carotid symptomatic plaques of a critical gene regulating inflammation and lesion stability, PLA2G7, which encodes lipoprotein-associated phospholipase A_2_ (LP-PLA_2_) [44,53]. Similarly, two studies have demonstrated the role of TET2 in reducing pro-inflammatory cytokine and chemokine expression as well as inflammasome activation, thus preventing atherosclerosis [54,55]. While TET2 has been related to antiatherosclerotic and vasoprotective roles [56], increased expression of TET1 in line with global DNA hypomethylation has been observed in advanced carotid atherosclerotic lesions compared to healthy arteries [57]. Of particular note is a counter mechanism for reversing DNA demethylation induced by TET, DNA methylation through DNA methyltransferase (DNMT). Increased DNMT1 in macrophages has been correlated with decreased peroxisome proliferator-activated nuclear receptor γ (PPAR-γ) and increased proinflammatory cytokines in an atherosclerosis-prone mice model as well as in atherosclerotic patients [43,58]. However, Xie et al. reported that DNMT1 expression was diminished in human atherosclerotic plaques [59]. In line with this, several studies have reported that DNMT1 and DNMT3a-dependent DNA methylation altered the blood flow, inducing atherosclerosis in murine models via modifications of transcriptional factors [57].

As has been mentioned, another factor that influences plaque vulnerability is its calcification status. It has recently been reported that in patients with carotid stenosis, highly-calcified plaques are associated with the hypomethylation of antiatherosclerotic genes such as receptor activity binding protein 1 (RAMP1), indicating that calcified atherosclerotic plaques are less vulnerable to rupture [60].

In summary, methylation patterns of promoter regions of a plethora of genes contributing to atherosclerosis undergo significant changes during the development of the disease, providing clear evidence that DNA methylation plays a significant concordant role in atherosclerotic plaque development and in progression towards vulnerable lesions [57], although the specific role still remains unclear.

### 3.2. Histone Post-Translational Modifications

Histone PTMs is another form of chromatin remodelling that has been described as playing a pivotal role in the activation and repression of gene transcription [43]. It is important to note that histone PTMs and DNA methylation interact to drive context-dependent gene transcription or repression [57]. Histone H2A, histone H2B, histone H3, and histone H4 can be modified by methylation, acetylation, ubiquitination, phosphorylation, SUMOylation, GlcNAcylation, carbonylation, and ADP-ribosylation, collectively constituting the histone code [43]. Histone acetylation generally promotes gene expression, whereas histone methylation promotes repression.

Changes in histone acetyl groups have been widely recognised as being epigenetic marks of atherosclerosis, since a correlation between histone acetylation and the atherosclerosis burden has been demonstrated in mice and humans [61,62]. Modulating histone acetyl transferases (HATs) have been linked to a reduction in the development of atherosclerosis in a mice model prone to develop this disease [42]. Additionally, histone deacetylase 3 (HDAC3) has been reported as having a protective effect in apolipoprotein E deficient (ApoE−/−) mice as it maintains the endothelial integrity and its deficiency results in atherosclerosis [22]. Similarly, patients with CAS showed enhanced histone acetylation as well as a methylation reduction in H3K9 and H3K27 in the smooth muscle cells from severe atherosclerotic lesions that correlated with plaque severity [22,43]. Moreover, euchromatic histone-lysine N-methyltransferase (EHMT2) is a histone methyltransferase that is responsible for the methylation of H3K9 and its depletion has been associated with the inhibition of the proliferation of endothelial cells, suggesting that this inhibition could suppress angiogenesis [57,63]. Indeed, the histone-lysine N-methyltransferase enzyme EZH2 has been strongly associated with atherosclerotic plaque development and vulnerability as it is actively involved in the transcription of pro-inflammatory genes [42,57]. In line with this, several histone demethylases or histone methyl transferases have been identified such as sirtuin 3 (SIRT3) and lysine-specific demethylase 7 homolog 3 (JMJD3) and associated with atherosclerosis development pathways including inflammation and endothelial functioning [57,64].

### 3.3. Non-Coding RNAs

A large part of the human genome is transcribed into ncRNAs, which have been viewed as potential biomarkers and therapeutic targets [43,65]. There are several subfamilies of ncRNAs such as microRNAs (miRNAs), long non-coding RNAs (lncRNAs), and circular RNAs (circRNAs) consisting of covalently closed continuous loops [43].

#### 3.3.1. miRNAs

miRNAs are endogenous small single-stranded ncRNAs around 22 nucleotides in length [66,67] that regulate gene expression at the post-transcriptional level by degrading target mRNAs or blocking their translation [66,68,69]. miRNAs are transcribed in the nucleus as pri-miRNAs, which are folded into hairpins and bound to the Drosha and Dicer enzymes. The Drosha forms a complex that cleaves the primary transcript and releases pre-miRNA, which is exported to the cytoplasm, where Dicer undergoes the maturation process to obtain two strands of miRNAs [67]. One of these two strands is assembled in the RNA-induced silencing complex (RISC), which is the cytoplasmatic effector, and can bind to the complementary sequence of the mRNA target [70]. The miRNAs’ seed sequence is 2–8 nucleotides at the 5′-untranslated end that would recognise and bind to the 3′-untranslated region of the target mRNA sequence [70,71]. Currently, there are 1917 annotated hairpin precursors and 2654 mature sequences in the human genome [72]. It is well-known that each miRNA can have multiple targets, and altogether, they regulate one third of the genes in the genome [73].

Due to their regulatory function, miRNAs play a significant role in diverse biological processes such as cell proliferation, differentiation, migration, and apoptosis [69,71,74]. Consequently, several studies have reported a close association between the dysregulation of miRNAs and human diseases [67,74], and emerging evidence suggests that miRNAs may be useful as diagnostic biomarkers [68]. The value of miRNAs is that they can be detected in blood, making it a non-invasive diagnostic biomarker that remains stable over time [75]. As discussed above, the identification of biomarkers that are able to predict vulnerable carotid plaques is crucial and, therefore, circulating miRNAs could be good candidates as they have already been shown to play an important role in the development of carotid stenosis [74,75].

Several studies have focused on identifying miRNA biomarkers of carotid stenosis by analysing differences in miRNA expression between CAS patients and healthy controls. For instance, miR-146a has been found to be higher expressed in the serum of CAS patients [76]. In this study, in which patients were also classified according to their degree of stenosis and plaque vulnerability, miR-146a, interleukin-6 (IL-6), and tumour necrosis factor alpha (TNF-α) expression levels positively correlated with both factors [76]. Luque et al. found a decrease in serum miR-638 levels in symptomatic and high-grade carotid stenosis patients, leading to ischaemic cerebrovascular events that could also be involved in plaque instability [77]. This particular miRNA inhibits the proliferation and migration of human vascular smooth muscle cells (VSMCs) by targeting the NOR1/cyclin D pathway [77]. Another study compared the expression of miRNAs previously related to adverse vascular remodelling between hypertensive patients with and without carotid plaque, describing hypertensive patients with plaques as having an increased serum expression of miR-145-5p and miR-let7c. These results suggest that these miRNAs potentially have a role as biomarkers for subclinical atherosclerosis in hypertensive individuals [78]. In addition, studies in mice showed an increased expression of miR-322-5p and a decreased expression of miR-206-3p in left carotid artery tissues of the atherosclerotic plaque group compared with the control group. miR-206-3p is involved in the phosphoinositide 3-kinase and protein kinase B (PI3K–AKT) pathway by regulating insulin-like growth factor 1 (Igf1), which has a role in maintaining plaque stability, and miR-322-5p interacts with mitogen-activated protein kinase 8 (MAPK8), whose loss accelerates the onset of early atherosclerosis [79].

On the other hand, some other studies have focused their efforts on identifying miRNAs that are differentially expressed between SCAS and ACAS patients. miR-100, miR-127, miR-125a, miR-133a, miR-145, miR-221, and miR-133b were higher expressed in symptomatic plaques than in asymptomatic ones [80,81,82]. These miRNAs are involved in vascular inflammation, apoptosis modulation, macrophage function, VSMCs differentiation, and plaque instability, among others [81]. However, for circulating miRNAs, we only found two studies that analysed differences between SCAS and ACAS patients. One study showed that SCAS patients had higher serum levels of miR-124-3p and miR-134-5p and lower levels of miR-133a-3p than ACAS patients [83]. These results seem to suggest that there is no correlation between plaque and the serum miR-133a expression profile among SCAS and ACAS patients [81,82,83]. The other study of circulating miRNAs did not provide strong evidence for associations between specific miRNAs, but there was a certain tendency showing higher plasma levels of miR-92a, miR-145, miR-210, and miR-143 in symptomatic versus asymptomatic patients [84]. Further work is needed to elucidate the role of circulating miRNAs between SCAS and ACAS patients.

Moreover, some other studies have found miRNAs that differentiate patients with ACAS and healthy individuals in order to diagnose them before the onset of symptoms. miR-186-5p showed an increased expression in the serum of ACAS patients [74], which may be involved in the development of atherosclerosis through the targeting of the PTEN/PI3K/AKT pathway [79,85]. miR-106-5p was also significantly upregulated in the serum of ACAS patients, which could regulate cholesterol efflux targeting ABCA1 [80,86]. miR-483-5p has also been found to be increased in the serum of ACAS patients versus healthy controls, and its underlying regulatory pathway could be the regulation of high cholesterol by targeting proprotein convertase subtilisin/kexin type 9 (PCSK9), affecting the development of ACAS [87]. Moreover, upregulation of miR-92a could also be a non-invasive serum biomarker for ACAS, which might be involved in the inflammatory response by enhancing nuclear factor kappa-light-chain-enhancer of activated B cells (NF-kB) [71,88] in the dysregulation of endothelial progenitor cells by targeting growth differentiation factor 11 (GDF11) via the SMAD2/3/FAK/AKT/eNOS pathway [71,89] and in the progression of atherosclerosis through the targeting of ABCA1 [71,90], as mentioned above for miR-106-5p. miR-92a has also shown higher plasma levels in symptomatic versus asymptomatic patients [84] as has been described in the previous paragraph, suggesting that the circulating concentration of miR-92a may gradually increase from healthy subjects to asymptomatic and symptomatic patients. Moreover, miR-342-5p was significantly overexpressed in asymptomatic serum patients, which positively correlates with IL-6 and TNFα and might be involved in the proliferation and differentiation of VSMCs downregulating PI3K regulatory subunit α (PI3KR1) [69,91]. On the other hand, miR-9-5p [75], miR-503-5p [92], and miR-28-5p [93] were significantly decreased in the serum of ACAS patients. A potential miR-28-5p target is forkhead box protein O1 (FOXO1), which stimulates the proliferation and migration of VSMCs in carotid artery stenosis [93]. In addition, the Kaplan–Meier method has been used to assess the predictive value of the occurrence of cerebrovascular ischaemic events (CIE) in some of the miRNAs discussed above. Multivariate Cox regression analysis suggests that altered serum levels of miR-186-5p [74], miR-106-5p [80], miR-483-5p [87], miR-92a [71], miR-342-5p [69], and miR-9-5p [75] could predict the risk of future cerebrovascular events. This would mean that they might also be valuable predictors of CIE and instability.

Another interesting area to study is the identification of miRNAs that discriminate between stable and vulnerable carotid plaques, which would allow for the identification of high and low risk patients and better selection in indicating surgical or intensive medical treatment. Plasma miRNAs such as miR-126 and miR-145 have been reported as being useful in predicting vulnerable plaque instability, as both were significantly associated with increased necrolipidic plaque tissue in patients with stable CAD [94]. Plasma miR-210 could stabilise carotid plaques by inhibiting adenomatous polyposis coli (APC), which is a well-known tumour suppressor gene with an inhibitory function in Wnt signalling, contributing to the balance between proliferation and apoptosis [95]. Furthermore, miR-199b-3p, miR-24-3p, miR-221-3p, miR-27b-3p, and miR-130a-3p were higher expressed in the plasma of patients with ACAS progression [74,96]. Other studies have reported that patients with vulnerable plaques had higher serum levels of miR-146a and lower serum levels of miR-320b [97] and miR-216b [98]. miR-320b networks with different biological pathways regulating its target proteins endothelin 1 (ET1), extracellular signal-regulated protein kinase 1 (ERK1), vascular endothelial growth factor (VEGF), and fibronectin (FN) [97,99]. With regard to miR-146a, it has also been previously reported as being increased in CAS patients compared to healthy controls [76], supporting the role of this miRNA in plaque development and destabilisation. It has also been demonstrated that circulating plasma miR-23a-5p is associated with macrophage-derived foam cell formation and might be a key regulator contributing to atherosclerotic plaque progression and vulnerability through negatively regulating cholesterol efflux in the ABCA1 and ABCG1-dependent pathway [100]. Another interesting miRNA is miR-21, whose role in atherosclerotic plaque progression has been well-established in ApoE-/-mice and exhibits lower miR-21 levels in vulnerable plaques [101,102]. Bazan et al. classified carotid stenosis patients as urgent, symptomatic, or asymptomatic according to the need for endarterectomy and the time elapsed. The results showed that the urgent group exhibited a significant decrease in miR-221 and miR-222 expression in the plaque shoulder, suggesting an association between these miRNAs and atherosclerotic plaque stability [103]. In contrast to these results, Maitrias et al. reported higher miR-221 levels in symptomatic versus asymptomatic plaques [82]. This discrepancy in miR-221 expression in symptomatic plaques may be due to different plaque regions being analysed (plaque shoulder versus entire plaque). In addition, miR-330-5p has also been reported as a differentially expressed miRNA in unstable plaques from patients with carotid stenosis, which may be playing an important role in plaque progression [16,71,104]. Other studies have reported that vulnerable plaques had higher levels of miR-200c in comparison to stable plaques [105]. Nie et al. established different spontaneous plaque rupture mice models and collected left carotid artery tissues at different time points from a control group, an atherosclerotic plaque group, and a plaque rupture group, reporting that miR-466h-5p is upregulated in the plaque rupture group and may promote atherosclerotic plaque rupture via apoptosis-related pathways [79]. In addition, the phenotypic change of macrophages to M1 is also a factor to consider in vulnerable plaques. Thus, it has also been shown that miR-216a could be promoting the shift to the M1 phenotype through the inhibition of SMAD family member 3 (SMAD3) and the consequent activation of telomerase reverse transcriptase (TERT) activity, which would entail atherosclerotic progression [106]. miR-532-3p has been found in silico analysis and validated in vitro as a direct negative regulator of the granulocyte-macrophage colony-stimulating factor 2 receptor subunit alpha (CSF2RA) transcription in humans and murine macrophages. CSF2RA is significantly upregulated in macrophage-rich vulnerable plaques promoting proinflammatory macrophage polarisation and inducing an atherogenic inflammatory response in the arterial wall. Therefore, macrophage deregulation of the miR-532-3p-CSF2RA axis is an important factor in vulnerable plaque progression [107]. Finally, as above-mentioned, stable carotid plaques tend to be more calcified, thus finding a miRNA profile of vascular calcification could also help us to predict its vulnerability. For instance, both miR-4530 and miR-133b are decreased in highly calcified carotid plaques, suggesting that these miRNAs may play a modulating role in calcification and plaque instability [108]. Different miRNA signatures can also distinguish between morphological subtypes of calcification in carotid atheromatous plaques, which may correspond to different stages in plaque progression. miR-30a-5p and miR-30d have been described as decreasing in calcific core (CC) plaques compared to protruding nodule (PN) plaques, which may reflect an acute stage of carotid pathology with a more active calcium turnover [109].

**Figure 1:**
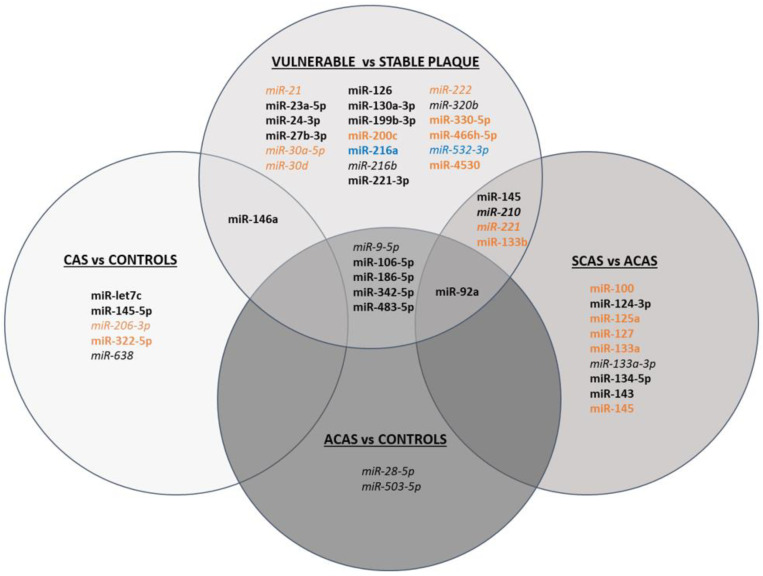
Schematic figure of miRNAs differentially expressed in CAS vs. controls, ACAS vs. controls, SCAS vs. ACAS, and vulnerable vs. stable plaques. miRNAs shown in black have been determined in serum/plasma, in orange in atherosclerotic plaque, and in blue in in vitro studies. Upregulated miRNAs are shown in bold, downregulated miRNAs in italics and miRNAs that have been found up- or downregulated depending on the study are shown in both bold and italics.

Figure 1 summarises all differentially expressed miRNAs according to their usefulness as potential biomarkers of carotid stenosis and/or plaque vulnerability.

#### 3.3.2. lncRNAs

lncRNAs are a family of non-protein coding transcripts that are longer than 200 nucleotides [57]. There are more than 100,000 lncRNAs in the human genome, which are classified into six categories based on their genomic locations including the dominant intergenic lncRNAs between two protein-coding genes, intronic lncRNAs in the introns of protein-coding genes, sense and antisense lncRNAs in the sense or antisense strands of protein-coding genes, bidirectional promoter lncRNAs in promoter regions, and enhancer lncRNAs in the enhancer regions of the genome [43]. A growing body of evidence supports lncRNAs playing critical roles in regulating multiple pathophysiological processes in CVDs [57], as most are present in the nucleus, recruiting epigenetic factors and triggering chromatin remodelling, thereby leading to the activation or repression of genes in the nucleus [43]. Compared to miRNAs, lncRNAs have more diverse functions as they act as chromatin regulators, miRNA sponge, decoy, guide, molecular scaffold, and enhancers [57]. However, understanding of the mechanisms by which lncRNAs control gene expression and regulate important cellular functions is still in its infancy [57].

In terms of vascular function, lncRNAs regulate endothelial function, involving proliferation, migration, apoptosis, and angiogenesis [57]. In line with this, the expression of five lncRNAs associated with CAD has been analysed in coronary tissue with and without atherosclerosis including cyclin-dependent kinase inhibitor 2B antisense RNA 1 (ANRIL), myocardial infarction associated transcript (MIAT), metastasis associated lung adenocarcinoma transcript 1 (MALAT1), KCNQ1 opposite strand/antisense transcript 1 (KCNQ1OT1), and HIF1A antisense RNA 2 (aHIF) although only three of them, ANRIL, MIAT, and MALAT1 displayed differences between atherosclerotic and non-atherosclerotic arteries [110]. Consistent with this, MIAT was markedly elevated in the serum and advanced carotid artery atherosclerotic plaques of patients with symptoms of vulnerable carotid atherosclerotic plaques as well as in serum and macrophages of necrotic cores in aortic atherosclerotic lesions of an advanced atherosclerosis mouse model [111,112] compared to non-atherosclerotic control arteries, mediating pro-inflammatory signalling through KLF4 [112]. Considering its critical role in regulating atherosclerotic plaque vulnerability, it has been reported that MIAT knockdown attenuates atherosclerosis progression, reduces necrotic core size, and increases plaque stability as well as promoting the clearance of apoptotic cells through sponging miR-149-5p in macrophages [111]. Another key regulator of cell proliferation and apoptosis during atherosclerosis preventing its development is lincRNA-p21, whose expression was dramatically downregulated in atherosclerotic plaques of ApoE -/-mice, leading to promoted cell proliferation and repressed apoptosis in VSMCs [113]. Specifically, lincRNA-p21 alleviates the progression of atherosclerosis progression by the mediation of the miR- 221/SIRT1/Pcsk9 axis [114]. Weng et al. reported the overexpression of the lncRNA LINC01123 in oxidised-LDL (ox-LDL)-induced VSMCs, promoting its migration and proliferation, and in the serum of CAS patients compared to healthy volunteers. LINC01123 modulates KLF5 expression through sponging miR-1277-5p to foster the migration and proliferation of VSMCs, thus promoting the formation of fibrous plaque, which is associated with stable plaque formation [115]. Additionally, lncRNA PEBP1P2 has also presented beneficial protective effects against abnormal VSCM proliferation and atherosclerosis, mainly by targeting the cyclin-dependent kinase 9 (CDK9) pathway. In accordance with this, its expression was decreased in serum of CAD patients and human advanced carotid atherosclerotic plaques as well as in a rat model of carotid artery injury [116]. With regard to inflammation, nuclear paraspeckle assembly transcript 1 (NEAT1) has been proposed as a candidate lncRNA involved in the attenuation of inflammation, as its overexpression is strongly associated with reduced TNF-α-induced vascular cell pro-inflammatory response and it has been found to be upregulated in peripheral blood mononuclear cells (PBMCs) from patients with CAD and in carotid artery atherosclerotic plaques [117]. A recent study has identified lncRNA AC009948.5, renamed as CHROME, in human plasma and carotid artery plaques, which harbours several binding sites for miRNAs that have been experimentally validated to regulate lipid metabolism and be related to plaque formation (i.e., miRN-27b, miR-33a and b, and miR-128) [118]. Additionally, another study of lncRNA UC.98, which is an lncRNA that is involved in atherosclerotic progression and postulated as a potential biomarker for the early diagnosis of atherosclerotic vulnerable plaques, described its overexpression in human blood of acute coronary syndrome (ACS) patients in vulnerable plaques [119]. Besides, two new lncRNAs, MSTRG.11455.17 and MSTRG.12845, have recently been described as regulating carotid plaque vulnerability as they have been found to be increased in unstable plaques in comparison to stable plaques and are involved in the control of energetic haemostasis and VSMCs proliferation and migration [120]. lncRNA CCAT2, whose role is well-known in tumours, has been studied in carotid plaque rupture and, interestingly, a positive correlation has been found between serum CCAT2 and plaque characteristics such as thickness [98]. Expression of serum CCAT2 is increased in patients with unstable carotid plaques, while miR-216b expression is lower, suggesting that CCAT2 and miR-216b could be used to predict the rupture of carotid atherosclerotic plaques [98]. Furthermore, a recent study comparing unstable vs. stable carotid atherosclerotic plaques identified a panel of 47 differentially expressed lncRNAs, from which LINC01272, also named PELATON, was upregulated in unstable carotid plaques [121]. PELATON is a nuclear expressed, monocyte- and macrophage-specific lncRNA enriched in areas of plaque inflammation that colocalise with the necrotic core of human plaque sections. It regulates cellular functions associated with plaque progression such as phagocytosis, lipid uptake, and/or reactive oxygen species production [121,122].

**Figure 2:**
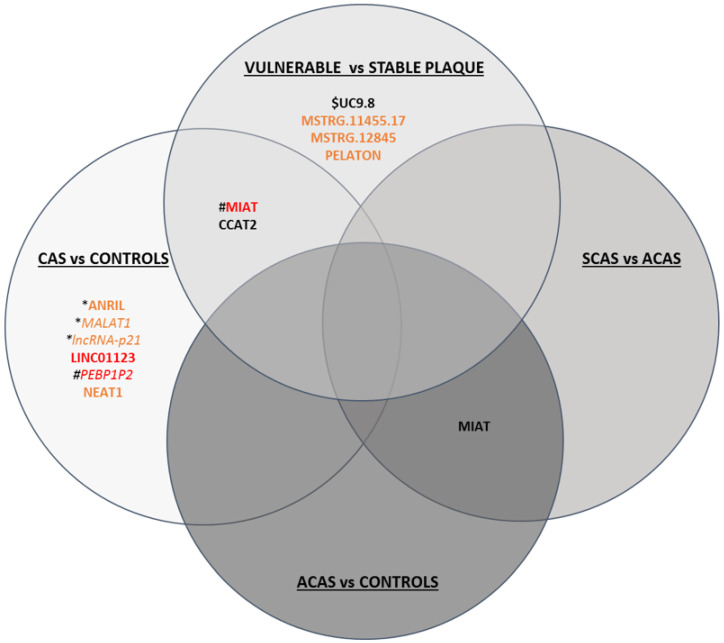
Schematic figure of lncRNAs differentially expressed in CAS vs. controls, ACAS vs. controls, SCAS vs. ACAS and vulnerable vs. stable plaques. LncRNAs shown in black have been determined in serum/plasma, in orange in plaque, and in red both in serum/plasma and plaque. Upregulated lncRNAs are in bold, while downregulated lncRNAs are in italics. * lncRNA dysregulated in coronary artery disease; # lncRNA dysregulated in carotid and coronary artery disease; $ lnCRNA dysregulated in acute coronary syndrome.

All differentially expressed lncRNAs related to atherosclerosis, carotid stenosis, and/or plaque vulnerability are summarised in Figure 2.

#### 3.3.3. circRNAs

circRNAs are pervasively expressed in mammalian tissues and often act as transcriptional/translational regulators or miRNA sponges [43]. These are a type of single-stranded RNA that form a covalently closed continuous loop, formed from pre-messenger RNAs by back-splicing [123]. The ring structure allows circRNAs to resist ribonuclease R activity and confers a longer half-life than that of linear RNAs [124].

**Figure 3:**
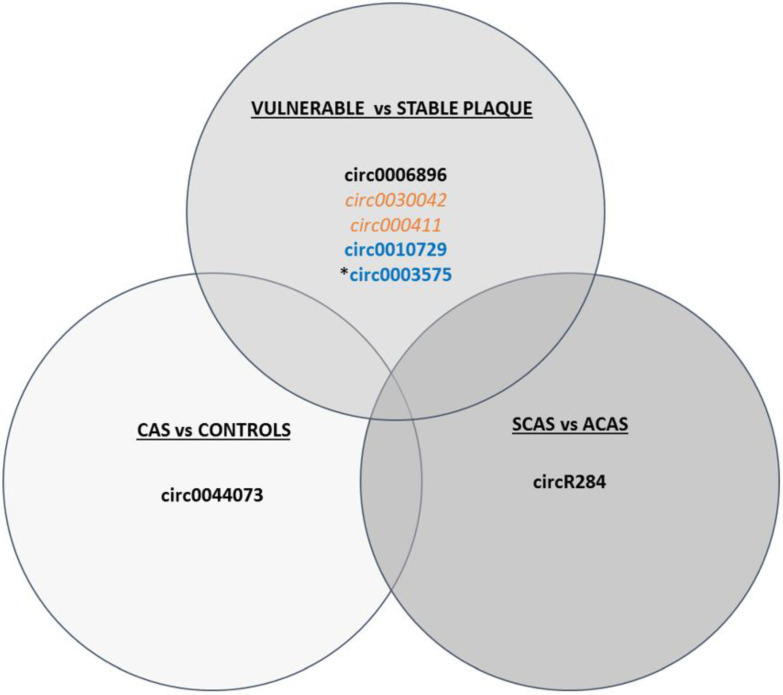
Schematic figure of circRNAs differentially expressed in CAS vs. controls, vulnerable vs. stable plaques, and SCAS vs. ACAS. circRNAs shown in black have been determined in serum/plasma, in orange in plaque, and in blue in in vitro studies. Upregulated circRNAs are in bold, while downregulated ones are in italics. * Pending validation studies.

Emerging studies have demonstrated that circRNAs are related to the development and progression of atherosclerosis [57]. For instance, recent studies have described a negative correlation between circular antisense non-coding RNA in the INK4 locus (circANRIL) and an increased risk of atherosclerotic vascular diseases in the artery plaques of both humans and animals by controlling ribosomal RNA (rRNA) maturation and modulating pathways of atherogenesis such as apoptosis induction and reduced proliferative capacity, conferring atheroprotection [125,126]. Human circ0003575 increases in response to ox-LDL-stimulation in human umbilical vein endothelial cells (HUVECs) but decreases when these cells proliferate, hypothesising that they regulate miR-199-3p, miR-9-5p, miR-377-3p, and miR-141-3p, which in turn control angiogenesis, cholesterol metabolism, VSMC proliferation, and migration, although further studies are needed to validate these associations [127]. In line with this, circ0030042 inhibits the ox-LDL-induced abnormal autophagy of HUVECs and maintains plaque stability in a mice model of atherosclerosis [124]. circ0044073 is another circRNA associated with atherosclerosis as it is upregulated in blood cells of carotid atherosclerotic patients and suppresses the levels of miR-107 via a sponge mechanism, which is involved in activating inflammatory, proliferative, and invasive pathways [128]. circR284 has also been identified as a potential inhibitor of miR-221 and miR-222 activity, which are involved in atherosclerotic plaque rupture [65]. In fact, an elevated ratio of serum circR284/miR-221 displayed favourable sensitivity and specificity for detecting plaque vulnerability and stroke, thus it has been postulated as a potential diagnostic biomarker of carotid plaque rupture [65]. Another example in the discrimination of patients with stable and unstable carotid plaques is circ0006896, which is increased in serum from patients with unstable/vulnerable plaques, and has been related to the regulation of miR-1264 and in turn DNMT1 expression, promoting the proliferation and migration of endothelial cells, thus playing an important role in carotid plaque destabilisation by regulating the behaviour of endothelial cells [129]. In agreement with this, circ000411 is downregulated in unstable carotid plaques compared to stable plaques and is involved in controlling apoptotic and autophagic pathways [120], suggesting that plaque vulnerability depends on different pathways and may be tackled from different perspectives. Furthermore, circ0010729 has been described as regulating vascular endothelial cell proliferation and apoptosis by targeting the miR-186/HIF-1α axis [130], adding another layer of complexity to epigenetic circuitry. All these differentially expressed circRNAs are summarised in Figure 3.

## 4. Limitations and Perspectives of the Epigenetic Biomarkers

Advances in methodology and big data analysis have identified epigenetic mechanisms as potential biomarkers of carotid atherosclerosis development and plaque vulnerability, which can be used in predicting stroke and possibly permitting more personalised diagnosis and treatment. However, there are a number of challenges and constraints that must be addressed before they can be applied in clinical practice. The lack of ethnic and geographical diversity among participants in published studies as well as of standardised clinical criteria to distinguish between ACAS patients (vulnerable vs. stable) are currently important biases. It is also necessary to confirm whether epigenetic biomarkers of vulnerable carotid plaque identified at tissue level are also differentially detected in blood and so are clinically useful for analysis by a minimally invasive method. Randomised and prospective multicentre studies with larger sample populations are required to determine with certainty which circulating epigenetic biomarkers are reliable for clinical routine in the identification of high-risk ACAS patients, and the management of asymptomatic carotid artery disease. These multicentre studies could also determine whether features of plaque vulnerability are interchangeable between vascular beds by evaluating whether high-risk markers validated in CAD are relevant in carotid plaque vulnerability, which could be relevant in the broad application of the vulnerable plaque concept in predicting and preventing CVDs [17].

Rather than a single epigenetic biomarker, a multimarker panel of epigenetic and other circulating biomarkers (e.g., protein-based) could improve the diagnostic and prognostic sensitivity and specificity of individual biomarkers of vulnerable carotid plaque. For a multimarker panel to be successful, it must provide additional information to the clinical diagnosis and give rapid results, and the instrumentation required must be easy to use and cost effective [23]. A risk score including the biomarkers, together with clinical data, should be used to risk stratify ACAS patients in further prospective trials. Such patients, in addition to conservative treatment (pharmacological treatment and levelling of modifiable risk factors) could be good candidates for a prophylactic interventional treatment (carotid endarterectomy or stenting) [14,24].

Among the epigenetic mechanisms, ncRNAs (miRNAs, lncRNAs and cirRNAs) are the most promising biomarkers for the risk stratification, diagnosis, and prognosis of various CVDs including vulnerable carotid atherosclerosis. In comparison with DNA methylation and histone PTMs, ncRNAs are considered as promising non-invasive biomarkers due to their tissue- and time-specific expression pattern in CVDs and their ability to circulate in the bloodstream in a relatively stable extracellular form [131]. Blood circulating miRNAs are the most studied. It has been found that the most circulating miRNAs in human plasma are related with Argonaute 2 (Ago2), a protein that forms a highly stabile complex that remains in the extracellular space [132,133]. The potential clinical utility of circulating lncRNAs and circRNAs as biomarkers is a burgeoning area of research. In particular, circRNAs are stable and abundant within exosomes and highly resistant to exonuclease RNase R, which makes them much more stable than linear RNAs in body fluids [129,131]. However, a global consensus needs to be reached on circulating ncRNA sampling, extraction, quantification, and data normalisation methods to ensure the accuracy and reproducibility of biomarker studies. With regard to sample preparation, it is highly recommended that serum or plasma be used instead of whole blood and that it be checked for haemolysis prior to processing and RNA isolation as it can alter the circulating ncRNA content [22,134,135]. While it is currently unclear whether serum or plasma should be used for the detection of circulating lncRNAs and circRNAs, plasma is the sample of choice for miRNA detection as the coagulation process may affect the spectrum of extracellular miRNAs in blood [136,137]. Regarding RNA extraction, several kits are commercially available with different extraction efficiencies of good quality and quantity for circulating ncRNAs. The quality of extracted ncRNAs is a crucial factor as it influences the sensitivity of downstream assays including quantitative reverse transcription-polymerase chain reaction (qRT-PCR) and microarray. Study protocols should therefore specify RNA extraction methods in order to ensure internal consistency and avoid problems with future reproducibility [136,138]. Circulating ncRNAs can be quantified, even at low amounts, with high sensitivity, specificity, and high dynamic range through qRT-PCR, a technique that is readily available at clinical laboratories [134,139]. Following quantification, data normalisation is critical to reduce technical variability in ncRNA quantification and is therefore necessary for rigorous biomarker investigation. It has been demonstrated that the choice of different normalisation strategies influences the results of gene expression analysis. The most common strategies used to normalise qRT-PCR data are based on (1) exogenous synthetic oligonucleotides (spike-in RNA) added to each sample following extraction; (2) geometrical mean of all the expressed ncRNAs; and (3) endogenous ncRNAs. However, spike-in normalisation does not consider internal variation in circulating ncRNAs between different individuals and the use of a single endogenous or reference gene is not sufficient to obtain reliable data. Therefore, it is important to select the most appropriate normalisation option for each experimental set and a combination of different normalisation methods should always be performed to guarantee the reliability of results [22,136,139,140,141,142]. Taken all together, the major drawback for using circulating ncRNAs as biomarkers in clinical practice is their laborious isolation and detection procedures. RNA isolation from serum or plasma and its subsequent quantification by qRT-PCR is expensive and time consuming. Therefore, it is important to find simpler, cheaper, and faster ways to detect circulating ncRNAs, which would make it available to clinical practice. To this end, new methodologies aimed at creating a point-of-care device for measuring ncRNAs rapidly are now being developed [143,144,145,146,147].

## 5. Conclusions

At present it is not possible to determine which carotid atherosclerotic plaques will become symptomatic and when. Although circulating protein-based biomarkers have been widely evaluated, there is a need for complementary biomarkers that may help in identifying patients with carotid vulnerable plaques that are high risk for stroke. Epigenetic biomarkers are gaining ground in the scientific community as tools for the diagnosis and prognosis of plaque vulnerability, and specifically, ncRNAs are promising biomarker candidates. To proceed to their clinical application, standardised pre-analytical, analytical, and post-analytical procedures should be agreed and large clinical studies, applying a combination of circulating biomarkers, together with clinical data and imaging biomarkers, will be essential to distinguish carotid plaques that may cause clinical symptoms of stroke and to advance in stroke prevention, thus decreasing the associated morbidity and mortality.

## Figures and Tables

**Table 1:** Clinical, haemodynamic, and neuroimaging parameters that are useful in routine clinical practice.

Greater Net Benefit if Revascularisation Performed	Lower Net Benefit if Revascularisation Performed
ICA stenosis >70%	ICA stenosis 50–69%
Male	Female
<70 years old	>75 years old
Diabetic	Not diabetic
Stroke	TIA or asymptomatic
Hemispheric symptoms	Amaurosis fugax
Plaque characteristics: hypoechoic, irregular surface, ulcerated, high volume, progression	Calcified plaque
Decreased CVR. Poor collateral circulation	Good collateral circulation
MES+	MES−

ICA: internal carotid artery. TIA: transient ischaemic attack. CVR: cerebrovascular reactivity. MES: microembolic signals.

## Supplementary Materials

The following supporting information can be downloaded at: https://www.mdpi.com/article/10.3390/ijms23095149/s1.
