# Supplementary material for: Clinical Parameters and Epigenetic Biomarkers of Plaque Vulnerability in Patients with Carotid Stenosis
Source: Int J Mol Sci. 2022 May 5;23(9):5149. doi: 10.3390/ijms23095149 (PMC9101730; doi:10.3390/ijms23095149)
Supplement: Supplementary file 1 [file ijms-23-05149-s001.zip › ijms-1684882-supplementary.pdf]

Supplementary Table S1. Epigenetic biomarkers for atherosclerosis, carotid stenosis and vulnerable plaque.

Epigenetic modification	*Biomarker*	*Regulation in atherosclerosis*	*Sample source and type*	*Reference*
**DNA methylation**	5-mC	Decreased in atherosclerotic vs. non-atherosclerotic aortic tissue and in SCAS vs. ACAS	Human and animal aortic tissue and human carotid plaques	[1,2]
	ESR1/2 promoter region	Hypermethylated in CAS vs. controls	Human blood samples and HUVEC	[1,3]
	ABCA1 promoter region	Hypermethylated is related to atherosusceptibility	HAEC and pig aortic Tissue	[4]
	KLF4 promoter region	Hypermethylated in CAS vs. controls,	Human carotid plaques	[4]
	AIRE1 and ALOX12 promoter regions	Hypomethylated in high-calcified vs. low-calcified carotid plaques	Human carotid plaques	[5]
	RAMP1 promoter region	Hypomethylated in CAS vs. controls	Human carotid plaques	[6]
	Inflammation genes [PLA2G7]	Hypomethylated in vulnerable plaques	Human carotid plaques and blood	[7]
	DNMT1	Increased in atherosclerosis	Human blood and mice aortic tissue	[8]
		It negatively regulates arterial stiffening via maintaining the contractile phenotype of VSMCs	VSMCs Culture	[9]
	TET1	Increased in CAS vs. controls	Humans carotid plaques	[10]
	TET2	Its loss correlates with the degree of injury in murine models of vascular injury and	Human blood and murine bone marrow macrophages and VSMCs culture	[11–13]

		human atherosclerotic disease		
**Histone PTMs**	HDAC3	Its deficiency results in atherosclerosis	Mice aortic tissue and aortic SMC culture	[14]
	H3K9 and H3K27 methylation	Decreased in carotid plaques and significantly associated with disease severity	Human and animal carotid plaques, cardiac and aortic tissue and plaque-derived VSMCs culture	[1,10,14,15]
	EZH2	Its overexpression exaggerates atherosclerotic lesion	Mice aortic Tissue	[10]
	SIRT3	Its deficiency impairs endothelium-dependent relaxation without affecting plaque instability	Mice aortic Tissue	[10,16]
	JMJD3	Its depletion attenuates expression of pro-inflammatory genes	Mice bone marrow-derived and peritoneal macrophages	[10]
**miRNAs**	miR-let7c	Increased in hypertensive patients with atherosclerotic plaque	Human Serum	[17]
	miR-9-5p	Decreased in ACAS vs. controls	Human Serum	[18]
		Decreased as a CIE predictor	Human Serum	[18]
	miR-21	Decreased in vulnerable plaque	Micro-dissected fibrous caps in mice plaques	[19,20]
	miR-23a-5p	Increased in vulnerable plaque	Human plasma	[21]

miR-24-3p	Increased in vulnerable plaque	Human plasma	[22]
miR-27b-3p	Increased in vulnerable plaque	Human plasma	[22]
miR-28-5p	Decreased in ACAS vs. controls	Human Serum	[23]
miR-30a-5p	Increased in PN plaques vs. CC plaques	Human carotid plaques	[24]
miR-30d	Increased in PN plaques vs. CC plaques	Human carotid plaques	[24]
miR-92a	Increased in ACAS vs. controls	Human Serum	[25]
	Increased in SCAS vs. ACAS*	Human plasma	[26]
	Increased as a CIE predictor	Human Serum	[25]
miR-100	Increased in SCAS vs. ACAS	Human carotid plaques	[27–29]
miR-106-5p	Increased in ACAS vs. controls	Human Serum	[27]
	Increased as a CIE predictor	Human Serum	[27]
miR-124-3p	Increased in SCAS vs. ACAS	Human Serum	[30]
miR-125a	Increased in SCAS vs. ACAS	Human carotid plaques	[27,28]
miR-126	Increased in vulnerable plaque	Human plasma	[31]
miR-127	Increased in SCAS vs. ACAS	Human carotid plaques	[27–29]
miR-130a-3p	Increased in vulnerable plaque	Human plasma	[22]

miR-133a	Increased in SCAS vs. ACAS	Human carotid plaque	[28,29]
miR-133a-3p	Decreased in SCAS vs. ACAS	Human Serum	[30]
miR-133b	Increased in SCAS vs. ACAS	Human carotid plaques	[28,29]
	Increased in vulnerable plaque	Human carotid plaques	[32]
miR-134-5p	Increased in SCAS vs. ACAS	Human Serum	[30]
miR-143	Increased in SCAS vs. ACAS*	Human plasma	[26]
miR-145	Increased in SCAS vs. ACAS	Human carotid plaques	[28,29]
	Increased in vulnerable plaque	Human plasma	[31]
	Increased in SCAS vs. ACAS*	Human plasma	[26]
miR-145-5p	Increased in hypertensive patients with atherosclerotic plaque	Human Serum	[17]
miR-146a	Increased in CAS vs. controls	Human Serum	[33]
	Increased in vulnerable plaque	Human Serum	[33]
miR-186-5p	Increased in ACAS vs. controls	Human Serum	[34]
	Increased as a CIE predictor	Human Serum	[34]

miR-199b-3p	Increased in vulnerable plaque	Human plasma	[22]
miR-200c	Increased in vulnerable plaque	Human carotid plaques	[35]
miR-206-3p	Decreased in CAS vs. controls	Mice left carotid artery tissue	[36]
miR-210	Decreased in vulnerable plaque	Human plasma sampled at the carotid lesion site	[37]
	Increased in SCAS vs. ACAS*	Human plasma	[26]
miR-216a	Increased in atherosclerosis progression	Human acute monocytic leukemia cell line	[38]
miR-216b	Decreased in unstable plaque	Human Serum	[39]
miR-221	Increased in SCAS vs. ACAS	Human carotid plaques	[28,29]
	Decreased in vulnerable plaque	Human carotid plaques shoulder	[40]
miR-221-3p	Increased in vulnerable plaque	Human plasma	[22]
miR-222	Decreased in vulnerable plaque	Human carotid plaques shoulder	[40]
miR-320b	Decreased in vulnerable plaque	Human Serum	[41]

	miR-322-5p	Increased in CAS vs. controls	Mice left carotid artery tissue	[36]
	miR-330-5p	Increased in vulnerable plaque	Human carotid plaques	[42]
	miR-342-5p	Increased in ACAS vs. controls	Human Serum	[43]
		Increased as a CIE predictor	Human Serum	[43]
	miR-466h-5p	Increased in vulnerable plaque	Mice left carotid artery tissue	[36]
	miR-483-5p	Increased in ACAS vs. controls	Human Serum	[44]
		Increased as a CIE predictor	Human Serum	[44]
	miR-503-5p	Decreased in ACAS vs. controls	Human Serum	[45]
	miR-532-3p	Decreased in vulnerable plaque	HEK-293T cells	[46]
	miR-638	Decreased in CAS vs. controls	Human Serum	[47]
	miR-4530	Increased in vulnerable plaque	Human carotid plaques	[32]
**lncRNA**	ANRIL	Increased in atherosclerosis vs. non-atherosclerosis	Human atherosclerotic coronary Plaques	[48]
	MIAT	Increased in atherosclerosis vs. non-atherosclerosis	Human atherosclerotic coronary plaque	[48]
		Increased in ACAS vs. controls and in SCAS vs. ACAS	Human serum	[49]

	Increased in atherosclerosis progression	Plaque and serum of an advanced atherosclerosis mouse model	[49]
	Increased in CAS vs. control and in vulnerable plaque	Human and murine carotid plaques	[50]
MALAT1	Decreased in atherosclerosis vs. non-atherosclerosis	Human atherosclerotic coronary plaque	[48]
lincRNA-p21	Decreased in atherosclerotic vs. non-atherosclerotic tissue	Mice aortic atherosclerotic plaques	[51]
LINC01123	Increased in CAS vs. controls	Human serum and carotid plaques	[52]
PEBP1P2	Decreased in atherosclerosis	Human serum of CAD patients and human advanced carotid plaques	[53]
NEAT1	Increased in CAS and CAD vs. controls	Human carotid plaques vs healthy arterial tissue and CAD PBMCs	[54]
CHROME	Increased in SCAS and ACAS vs. controls and in CAD vs controls	Human carotid plaques and plasma from CAD patients	[55]
UC.98	Increased in vulnerable plaques and involved in atherosclerotic progression	Human blood from ACS patients and MAEC culture	[56]
MSTRG.11455.17	Increased in unstable vs. stable plaques	Human carotid plaques	[57]
MSTRG.12845	Increased in unstable vs. stable plaques	Human carotid plaques	[57]

	CCAT2	Increased in unstable vs. stable plaques and vs. controls	Human serum of patients with carotid plaques	[39]
	PELATON	Increased in unstable vs. stable plaques	Human carotid plaques	[58]
**circRNA**	circANRIL	Increased in human vascular tissue	Human carotid plaques	[59]
		Its inhibition reduces vascular endothelial injury, oxidative stress and inflammation	Rat coronary artery tissue	[60]
	circ0003575	Increased in oxidized LDL treated cells	HUVEC culture	[61]
	circ0030042	Decreased in vulnerable plaques	Mice aortic root plaques	[62]
	circ0044073	Upregulated in CAS vs. controls	Human blood cells	[63]
	circR284	Increased in SCAS vs. ACAS	Human Serum	[64]
	circ0006896	Increased in unstable vs. stable plaques	Human serum exosomes of patients with carotid plaques	[65]
	circ000411	Decreased in unstable vs. stable plaques	Human carotid plaques	[57]
	circ0010729	Increased in dysfunctional vascular endothelial cells	HUVEC culture	[66]

ACAS: asymptomatic carotid artery stenosis. ACS: acute coronary syndrome. CAD: coronary artery disease. CAS: carotid artery stenosis. CIE: cerebrovascular ischaemic event. HAEC: human aortic endothelial cells. HUVEC: human umbilical vein endothelial cells. MAEC: murine aortic endothelial cells. SCAS: symptomatic carotid artery stenosis. SCM: smooth muscle cells. VSMCs: vascular smooth muscle cells. *Not statistically significant, a tendency to be validated.

References

1. Zhang, H.; Lang, Z.; Zhu, J.K. Dynamics and function of DNA methylation in plants. Nat Rev Mol Cell Biol 2018, 19, 489–506.
2. Zaina S. Unraveling the DNA methylome of atherosclerosis. Curr Opin Lipidol 2014, 25, 148–153.

3. Zaina, S.; Heyn, H.; Carmona, F.J.; Varol, N.; Sayols, S.; Condom, E.; Ramírez-Ruz, J.; Gomez, A.; Gonçalves, I.; Moran, S.; Esteller, M. DNA methylation map of human atherosclerosis. Circ Cardiovasc Genet 201, 7, 692–700.
4. Jiang, Y.Z.; Jiménez, J.M.; Ou, K.; McCormick, M.E.; Zhang, L.D.; Davies, P.F. Hemodynamic disturbed flow induces differential DNA methylation of endothelial Kruppel-like factor 4 promoter in vitro and in vivo. Circ Res 2014, 115, 32–43.
5. Kim, J.Y.; Choi, B.G.; Jelinek, J.; Kim, D.H.; Lee, S.H.; Cho, K.; Rha, S.H.; Lee, Y.H.; Jin, H.S.; Choi, D.K.; Kim, G.E.; Kwon, S.U.; Hwang, J.; Cha, J.K.; Lee, S.; Issa, J.J.; Kim, J. Promoter methylation changes in ALOX12 and AIRE1: Novel epigenetic markers for atherosclerosis. Clin Epigenetics 2020, 12, 66.
6. Katano, H.; Mase, M. Epigenetic changes in carotid plaques with high calcium scores; DNA methylation and microrna assessments. Atherosclerosis 2021, 331, e93.
7. Li, J.; Zhang, X.; Yang, M.; Yang, H.; Xu, N.; Fan, X.; Liu, G.; Jiang, X.; Fan, J.; Zhang, L.; Zhang, H.; Zhou, Y.; Li, R.; Gao, S.; Jin, J.; Jin, Z.; Zheng, J.; Tu, Q.; Ren, J. DNA methylome profiling reveals epigenetic regulation of lipoprotein-associated phospholipase A2 in human vulnerable atherosclerotic plaque. Clin Epigenetics 2021, 13, 161.
8. Yu, J.; Qiu, Y.; Yang, J.; Bian, S.; Chen, G.; Deng, M.; Kang, H.; Huang, L. DNMT1-PPARγ pathway in macrophages regulates chronic inflammation and atherosclerosis development in mice. Sci Rep 2016, 6.
9. Xie, S.A.; Zhang, T.; Wang, J.; Zhao, F.; Zhang, Y.P.; Yao, W.J.; Hur, S.S.; Yeh, Y.T.; Pang, W.; Zheng, L.S.; Fan, Y.B.; Kong, W.; Wang, X.; Chiu, J.J.; Zhou, J. Matrix stiffness determines the phenotype of vascular smooth muscle cell in vitro and in vivo: Role of DNA methyltransferase 1. Biomaterials 2018, 155, 203–16.
10. Xu, S.; Kamato, D.; Little, P.J.; Nakagawa, S.; Pelisek, J.; Jin, Z.G. Targeting epigenetics and non-coding RNAs in atherosclerosis: from mechanisms to therapeutics. Pharmacol Ther 2019, 196, 15–43.
11. Jaiswal, S.; Natarajan, P.; Silver, A.J.; Gibson, C.J.; Bick, A.G.; Shvartz, E.; McConkey, M.; Gupta, N.; Gabriel, S.; Ardissino, D.; Baber, U.; Mehran, R.; Fuster, V.; Danesh, J.; Frossard, P.; Saleheen, D.; Melander, O.; Sukhova, G.K.; Neuberg, D.; Libby, P.;…Ebert, B.L. Clonal Hematopoiesis and Risk of Atherosclerotic Cardiovascular Disease. N Engl J Med 2017, 377, 111–121.
12. Fuster, J.J.; MacLauchlan, S.; Zuriaga, M.A.; Polackal, M.N.; Ostriker, A.C.; Chakraborty, R.; Wu, C.L.; Sano, S.; Muralidharan, S.; Rius, C.; Vuong, J.; Jacob, S.; Muralidhar, V.; Robertson, A.A.; Cooper, M.A.; Andrés, V.; Hirschi, K.K.; Martin, K.A.; Walsh, K. Clonal hematopoiesis associated with TET2 deficiency accelerates atherosclerosis development in mice. Science 2017, 355, 842–847.
13. Liu, R.; Jin, Y.; Tang, W.H.; Qin, L.; Zhang, X.; Tellides, G.; Hwa, J.; Yu, J.; Martin, K.A. Ten-eleven translocation-2 (TET2) is a master regulator of smooth muscle cell plasticity. Circulation 2013, 128, 2047–2057.
14. Soler-Botija, C.; Gálvez-Montón, C.; Bayés-Genís, A. Epigenetic Biomarkers in Cardiovascular Diseases. Front Genet 2019, 10, 1–31.
15. Greißel, A.; Culmes, M.; Burgkart, R.; Zimmermann, A.; Eckstein, H.-H.; Zernecke, A.; Pelisek, J. Histone acetylation and methylation significantly change with severity of atherosclerosis in human carotid plaques. Cardiovasc Pathol 2016, 25, 79–86.
16. Winnik, S.; Gaul, D.S.; Preitner, F.; Lohmann, C.; Weber, J.; Miranda, M.X.; Liu, Y.; van Tits, L.J.; Mateos, J.M.; Brokopp, C.E.; Auwerx, J.; Thorens, B.; Lüscher, T.F.; Matter, C.M. Deletion of Sirt3 does not affect atherosclerosis but accelerates weight gain and impairs rapid metabolic adaptation in LDL receptor knockout mice: Implications for cardiovascular risk factor development. Basic Res Cardiol 2014, 109, 399 .
17. Minin, E.O.Z.; Paim, L.R.; Lopes, E.C.P.; Bueno, L.C.M.; Carvalho-Romano, L.F.R.S.; Marques, E.R.; Vegian, C.; Pio-Magalhaes, J.A.; Coelho-Filho, O.R.; Sposito, A.C.; Matos-Souza, J.R.; Nadruz, W.; Schreiber, R. Association of circulating mir-145-5p and mir-let7c and atherosclerotic plaques in hypertensive patients. Biomolecules 2021, 11, 1840 .
18. Liu, H.; Zhou, J.; Jiang, W.; Wang, F. Analysis of the diagnostic and prognostic value of miR-9-5p in carotid artery stenosis. Bosn J Basic Med Sci 2021, 21, 723–728.
19. Jin, H.; Li, D.Y.; Chernogubova, E.; Sun, C.; Busch, A.; Eken, S.M.; Saliba-Gustafsson, P.; Winter, H.; Winski, G.; Raaz, U.; Schellinger, I.N.; Simon, N.; Hegenloh, R.; Matic, L.P.; Jagodic, M.; Ehrenborg, E.; Pelisek, J.; Eckstein, H.H.; Hedin, U.; Backlund, A.;…Maegdefessel, L. Local Delivery of miR-21 Stabilizes Fibrous Caps in Vulnerable Atherosclerotic Lesions. Mol Ther 2018, 26, 1040–1055.
20. Kumric, M.; Borovac, J.A.; Martinovic, D.; Kurir, T.T.; Bozic, J. Circulating biomarkers reflecting destabilization mechanisms of coronary artery plaques: Are we looking for the impossible?. Biomolecules 2021, 11, 881 .

21. Yang, S.; Ye, Z.; Chen, S.; Luo, X.; Chen, S.; Mao, L.; Li, Y.; Jin, H.; Yu, C.; Xiang, F.X.; Xie, M.X.; Chang, J.; Xia, Y.P.; Hu, B. MicroRNA-23a-5p promotes atherosclerotic plaque progression and vulnerability by repressing ATP-binding cassette transporter A1/G1 in macrophages. J Mol Cell Cardiol 2018, 123, 139–149.
22. Dolz, S.; Górriz, D.; Tembl, J.I.; Sánchez, D.; Fortea, G.; Parkhutik, V.; Lago, A. Circulating MicroRNAs as novel biomarkers of stenosis progression in asymptomatic carotid stenosis. Stroke 2017, 48, 10–16.
23. Liu, Q.; Yan, S.; Yuan, Y.; Ji, S.; Guo, L. miR-28-5p improved carotid artery stenosis by regulating vascular smooth muscle cell proliferation and migration. Vascular 2021.
24. Vasuri, F.; Ciavarella, C.; Fittipaldi, S.; Pini, R.; Vacirca, A.; Gargiulo, M.; Faggioli, G.; Pasquinelli, G. Different histological types of active intraplaque calcification underlie alternative miRNA-mRNA axes in carotid atherosclerotic disease. Virchows Arch 2020, 476, 307–316.
25. Chen, G.; Gao, J.; Sheng, Y.; Han, X.; Ji, X.; Zhao, M.; Wu, J. Diagnostic value of miR-92a in asymptomatic carotid artery stenosis patients and its ability to predict cerebrovascular events. Diagn Pathol 2020, 15, 1–8.
26. Grosse, G.M.; Derda, A.A.; Stauss, R.D.; Neubert, L.; Jonigk, D.D.; Kühnel, M.P.; Gabriel, M.M.; Schuppner, R.; Wilhelmi, M.; Bär, C.; Bauersachs, J.; Schrimpf, C.; Thum, T.; Weissenborn, K. Circulating microRNAs in Symptomatic and Asymptomatic Carotid Stenosis. Front Neurol 2021, 12, 755827.
27. Zhang, T.; Chen, Z.; Yang, X.; Fu, R.; Wang, J.; Xu, H. Circulating miR-106b-5p serves as a diagnostic biomarker for asymptomatic carotid artery stenosis and predicts the occurrence of cerebral ischemic events. Vasc Med (United Kingdom) 2020, 25, 436–442.
28. Maitrias, P.; Metzinger-Le Meuth, V.; Massy, ZA.; M'Baya-Moutoula, E.; Reix, T.; Caus, T.; Metzinger, L. MicroRNA deregulation in symptomatic carotid plaque. J Vasc Surg 2015, 62, 1245-1250.
29. Cipollone, F.; Felicioni, L.; Sarzani, R.; Ucchino, S.; Spigonardo, F.; Mandolini, C.; Malatesta, S.; Bucci, M.; Mammarella, C.; Santovito, D.; de Lutiis, F.; Marchetti, A.; Mezzetti, A.; Buttitta, F. A unique MicroRNA signature associated with plaque instability in humans. Stroke 2011, 42, 2556–2563.
30. Badacz, R.; Przewłocki, T.; Gacoń, J.; Stępień, E.; Enguita, F.J.; Karch, I.; Zmudk, K.; Kablak-Ziembicka, A. Circulating miRNA levels differ with respect to carotid plaque characteristics and symptom occurrence in patients with carotid artery stenosis and provide information on future cardiovascular events. Adv Interv Cardiol 2018, 14, 75–84.
31. Knoka, E.; Trusinskis, K.; Mazule, M.; Briede, I.; Crawford, W.; Jegere, S.; Kumsars, I.; Narbute, I.; Sondore, D.; Lejnieks, A.; Erglis, A. Circulating plasma microRNA-126, microRNA-145, and microRNA-155 and their association with atherosclerotic plaque characteristics. J Clin Transl Res 2019, 5, 60–67.
32. Katano, H.; Nishikawa, Y.; Yamada, H.; Yamada, K.; Mase, M. Differential Expression of microRNAs in Severely Calcified Carotid Plaques. J Stroke Cerebrovasc Dis 2018, 27, 108–117.
33. Huang, P.; He, X.Y.; Xu, M. The Role of miRNA-146a and Proinflammatory Cytokines in Carotid Atherosclerosis. Biomed Res Int 2020, 2020, 6657734.
34. Lv, W.; Zhang, T.; Zhao, H.; He, S.; Li, B.; Gao, Y.; Pan, W. Diagnostic value of miR-186-5p for carotid artery stenosis and its predictive significance for future cerebral ischemic event. Diagn Pathol 2020, 15, 1–8.
35. Magenta, A.; Sileno, S.; D'Agostino, M.; Persiani, F.; Beji, S.; Paolini, A.; Camili, D.; Platone, A.; Capogrossi, M.C.; Fugiuele, S. Atherosclerotic plaque instability in carotid arteries: MiR-200c as a promising biomarker. Clin Sci 2018, 132, 2423–2436.
36. Nie, P.; Yang, F.; Wan, F.; Jin, S.; Pu, J. Analysis of MicroRNAs Associated With Carotid Atherosclerotic Plaque Rupture With Thrombosis. Front Genet 2021, 12, 1–15.
37. Eken, S.M.; Jin, H.; Chernogubova, E.; Li, Y.; Simon, N.; Sun, C.; Korzunowicz, G.; Busch, A.; Bäcklund, A.; Österholm, C.; Razuvaev, A.; Renné, T.; Eckstein, H.H.; Pelisek, J.; Eriksson, P.; González Díez, M.; Perisic Matic, L.; Schellinger, I.N.; Raaz, U.; Leeper, N.J.;…Maegdefessel, L. MicroRNA-210 enhances fibrous cap stability in advanced atherosclerotic lesions. Circ Res 2017, 120, 633–644.
38. Yang, S.; Li, J.; Chen, Y.; Zhang, S.; Feng, C.; Hou, Z.; Cai, J.; Wang, Y.; Hui, R.; Lv, B.; Zhang, W. MicroRNA-216a promotes M1 macrophages polarization and atherosclerosis progression by activating telomerase via the Smad3/NF-κB pathway. Biochim Biophys Acta - Mol Basis Dis 2019, 1865, 1772–1781.
39. Huang, C.-Q.; Jin, W.-X.; YU, G.-F. Correlation between carotid atherosclerotic plaque properties and serum levels of lncRNA CCAT2 and miRNA-216b. Eur Rev Med Pharmacol Sci 2020, 24, 7033–7038.

40. Bazan, H.A.; Hatfield, S.A.; O'Malley, C.B.; Brooks, A.J.; Lightell, D.; Woods, T.C. Acute Loss of MIR-221 and MIR-222 in the Atherosclerotic Plaque Shoulder Accompanies Plaque Rupture. Stroke 2015, 46, 3285–3287.
41. Zhang, R.; Qin, Y.; Zhu, G.; Li, Y.; Xue, J. Low serum miR-320b expression as a novel indicator of carotid atherosclerosis. J Clin Neurosci 2016, 33, 252–258.
42. Wei, X.; Sun, Y.; Han, T.; Zhu, J.; Xie, Y.; Wang, S.; Wu, Y.; Fan, Y.; Sun, X.; Zhou, J.; Zhao, Z.; Jing, Z. Upregulation of miR-330-5p is associated with carotid plaque's stability by targeting Talin-1 in symptomatic carotid stenosis patients. BMC Cardiovasc Disord 2019, 19, 1–8.
43. Zhou, A.; Li, Y.; Wang, P.; Yu, P.; Lang, L. Circulating miR-342-5p serves as a diagnostic biomarker in patients with carotid artery stenosis and predicts the occurrence of the cerebral ischemic event. Ir J Med Sci 2021, 191, 713-718.
44. Li, R.; Jiang, L.; Wang, X. Aberrant expression of miR-483-5p in patients with asymptomatic carotid artery stenosis and its predictive value for cerebrovascular event occurrence. Exp Ther Med 2021, 22, 1–7.
45. Yan, Z.; Wang, H.; Liang, J.; Li, Y.; Li, X. MicroRNA-503-5p improves carotid artery stenosis by inhibiting the proliferation of vascular smooth muscle cells. Exp Ther Med 2020, 20, 85.
46. Huang, R.; Cao, Y.; Li, H.; Hu, Z.; Zhang, H.; Zhang, L.; Su, W.; Xu, Y.; Liang, L.; Melgiri, N.D.; Jiang, L.; Li, X. miR-532-3p-CSF2RA Axis as a Key Regulator of Vulnerable Atherosclerotic Plaque Formation. Can J Cardiol 2020, 36, 1782–1794.
47. Luque, A.; Farwati, A.; Krupinski, J.; Aran, J.M. Association between low levels of serum miR-638 and atherosclerotic plaque vulnerability in patients with high-grade carotid stenosis. J Neurosurg 2019, 131, 72–79.
48. Arslan, S.; Berkan, Ö.; Lalem, T.; Özbilüm, N.; Göksel, S.; Korkmaz, Ö.; Çetin, N.; Devaux, Y. Long non-coding RNAs in the atherosclerotic plaque. Atherosclerosis 2017, 266, 176–181.
49. Ye, Z.M.; Yang, S.; Xia, Y.P.; Hu, R.T.; Chen, S.; Li, B.W.; Chen, S.L.; Luo, X.Y.; Mao, L.; Li, Y.; Jin, H.; Qin, C.; Hu, B. LncRNA MIAT sponges miR-149-5p to inhibit efferocytosis in advanced atherosclerosis through CD47 upregulation. Cell Death Dis 2019, 10, 138.
50. Fasolo, F.; Jin, H.; Winski, G.; Chernogubova, E.; Pauli, J.; Winter, H.; Li, D.; Glukha, N.; Bauer, S.; Metschl, S.; Wu, Z.; Koschinsky, M.L.; Reilly, M.; Pelisek, J;. Kempf, W.; Eckstein, H.H.; Soehnlein, O.; Matic, L.; Hedin, U.; Bäcklund, A.;…Maegdefessel, L. Long Noncoding RNA MIAT Controls Advanced Atherosclerotic Lesion Formation and Plaque Destabilization. Circulation 2021, 144, 1567–1583.
51. Wu, G.; Cai, J.; Han, Y.; Chen, J.; Huang, Z.P.; Chen, C.; Cai, Y.; Huang, H.; Yang, Y.; Liu, Y.; Xu, Z.; He, D.; Zhang, X.; Hu, X.; Pinello, L.; Zhong, D.; He, F.; Yuan, G.C.; Wang, D.Z.; Zeng, C. LincRNA-p21 regulates neointima formation, vascular smooth muscle cell proliferation, apoptosis, and atherosclerosis by enhancing p53 activity. Circulation 2014, 130, 1452–1465.
52. Weng, G.; Gu, M.; Zhang, Y.; Zhao, G.; Gu, Y. LINC01123 promotes cell proliferation and migration via regulating miR-1277-5p/KLF5 axis in ox-LDL-induced vascular smooth muscle cells. J Mol Histol 2021, 52, 943–953.
53. He, X.; Lian, Z.; Yang, Y.; Wang, Z.; Fu, X.; Liu, Y.; Li, M.; Tian, J.; Yu, T.; Xin, H. Long Non-coding RNA PEBP1P2 Suppresses Proliferative VSMCs Phenotypic Switching and Proliferation in Atherosclerosis. Mol Ther - Nucleic Acids 2020, 22, 84–98.
54. Vlachogiannis, N.I.; Sachse, M.; Georgiopoulo, S. ; Zormpas, E.; Bampatsias, D.; Delialis, D.; Bonini, F.; Glayfos, G.; Sigala, F.; Stamatelopoulos, K.; Gatsiou, A.; Stellos, K. Adenosine-to-inosine Alu RNA editing controls the stability of the pro-inflammatory long noncoding RNA NEAT1 in atherosclerotic cardiovascular disease. J Mol Cell Cardiol 2021, 160, 111–120.
55. Hennessy, E.J.; van Solingen, C.; Scacalossi, K.R.; Ouimet, M.; Afonso, M.S.; Prins, J.; Koelwyn, G.J.; Sharma, M.; Ramkhelawon, B.; Carpenter, S.; Busch, A.; Chernogubova, E.; Matic, L.P.; Hedin, U.; Maegdefessel, L.; Caffrey, B.E.; Hussein, M.A.; Ricci, E.P.; Temel, R.E.; Garabedian, M.J.;…Moore, K.J. The long noncoding RNA CHROME regulates cholesterol homeostasis in primates. Nat Metab 2019, 1, 98–110.
56. Fan, Z.; Zhang, Y.; Xiao, D.; Ma, J.; Liu, H.; Shen, L.; Zhang, M.; He, B. Long noncoding RNA UC.98 stabilizes atherosclerotic plaques by promoting the proliferation and adhesive capacity in murine aortic endothelial cells. Acta Biochim Biophys Sin (Shanghai) 2020, 52, 141–149.
57. Bao, M.H.; Zhang, R.Q.; Huang, X.S.; Zhou, J.; Guo, Z.; Xu, B.F.; Liu, R. Transcriptomic and Proteomic Profiling of Human Stable and Unstable Carotid Atherosclerotic Plaques. Front Genet 2021, 12, 755507.
58. Hung, J.; Scanlon, J.P.; Mahmoud, A.D.; Rodor, J.; Ballantyne, M.; Fontaine, M.; Temmerman, L.; Kaczynski, J.; Connor, K.L.; Bhushan, R.; Biessen, E.; Newby, D.E.; Sluimer, J.C.; Baker ,A.H. Novel

plaque enriched long noncoding RNA in atherosclerotic macrophage regulation (PELATON). Arterioscler Thromb Vasc Biol 2020, 40, 697–713.

59. Holdt, L.M.; Stahringer, A.; Sass, K.; Pichler, G.; Kulak, N.A.; Wilfert, W.; Kohlmaier, A.; Herbst, A.; Northoff, B.H.; Nicolaou, A.; Gäbel, G.; Beutner, F.; Scholz, M.; Thiery, J.; Musunuru, K.; Krohn, K.; Mann, M.; Teupser, D. Circular non-coding RNA ANRIL modulates ribosomal RNA maturation and atherosclerosis in humans. Nat Commun 2016, 7, 12429.
60. Shi, P.; Ji, H.; Zhang, H.; Yang, J.; Guo, R.; Wang, J. circANRIL reduces vascular endothelial injury, oxidative stress and inflammation in rats with coronary atherosclerosis. Exp Ther Med 2020, 20, 2245-2251.
61. Li, C.Y.; Ma, L.; Yu, B. Circular RNA hsa_circ_0003575 regulates oxLDL induced vascular endothelial cells proliferation and angiogenesis. Biomed Pharmacother 2017, 95, 1514–1519.
62. Yu, F.; Zhang, Y.; Wang, Z.; Gong, W.; Zhang, C. Hsa_circ_0030042 regulates abnormal autophagy and protects atherosclerotic plaque stability by targeting eIF4A3. Theranostics 2021, 11, 5404–5417.
63. Shen, L.; Hu, Y.; Lou, J.; Yin, S.; Wang, W.; Wang, Y.; Xia, Y.; Wu, W. CircRNA-0044073 is upregulated in atherosclerosis and increases the proliferation and invasion of cells by targeting miR-107. Mol Med Rep 2019, 49, 3923–39232.
64. Bazan, H.A.; Hatfield, S.A.; Brug, A.; Brooks, A.J.; Lightell, D.J.; Woods, T.C. Carotid Plaque Rupture Is Accompanied by an Increase in the Ratio of Serum circR-284 to miR-221 Levels. Circ Cardiovasc Genet 2017, 10, e001720 .
65. Wen, Y.; Chun, Y.; Qing Lian, Z.; Wei Yong, Z.; Mei Lan, Y.; Huan, L.; Xi, C.Y.; Juan, L.S.; Qing, Z.W.; Jia, C.; Ji, Z.H. circRNA-0006896-miR1264-DNMT1 axis plays an important role in carotid plaque destabilization by regulating the behavior of endothelial cells in atherosclerosis. Mol Med Rep 2021, 23, 311.
66. Dang, R.Y.; Liu, F.L.; Li, Y. Circular RNA hsa_circ_0010729 regulates vascular endothelial cell proliferation and apoptosis by targeting the miR-186/HIF-1α axis. Biochem Biophys Res Commun 2017, 490, 104–110.
